# 

**Published:** 1842-10-01

**Authors:** 


Published EVERY SATURDAY by J. C. TURNPENNY, N. E. corner of Spruce and Tenth streets.

